# Numerical Simulation of Entropy Generation for Power-Law Liquid Flow over a Permeable Exponential Stretched Surface with Variable Heat Source and Heat Flux

**DOI:** 10.3390/e21050484

**Published:** 2019-05-10

**Authors:** Mohamed Abd El-Aziz, Salman Saleem

**Affiliations:** 1 Department of Mathematics, College of Sciences, King Khalid University, Abha 61413, Saudi Arabia; 2 Department of Mathematics, Faculty of Science, Helwan University, Helwan-Cairo 11795, Egypt

**Keywords:** entropy generation, power-law fluid, non-uniform heat source, permeable surface, numerical scheme

## Abstract

This novel work explored the second law analysis and heat transfer in a magneto non-Newtonian power-law fluid model with the presence of an internal non-uniform heat source/sink. In this investigation, the motion of the studied fluid was induced by an exponentially stretching surface. The rheological behavior of the fluid model, including the shear thinning and shear thickening properties, are also considered as special case studies. The physical problem developed meaningfully with the imposed heat flux and the porosity of the stretched surface. Extensive numerical simulations were carried out for the present boundary layer flow, in order to study the influence of each control parameter on the boundary layer flow and heat transfer characteristics via various tabular and graphical illustrations. By employing the Shooting Runge–Kutta–Fehlberg Method (SRKFM), the resulting nonlinear ordinary differential equations were solved accurately. Based on this numerical procedure, the velocity and temperature fields are displayed graphically. By applying the second law of thermodynamics, and characterizing the entropy generation and Bejan number, the present physical problem was examined and discussed thoroughly in different situations. The attained results showed that the entropy generation can be improved significantly by raising the magnetic field strength and the group parameter. From an energetic point of view, it was found that the Reynolds number boosts the entropy generation of the fluidic medium and reduces the Bejan number. Also, it was observed that an amplification of the power-law index diminished the entropy generation near the stretched surface. As main results, it was proven that the heat transfer rate can be reduced with both the internal heat source intensity and the magnetic field strength.

## 1. Introduction

The collective necessity of energy with controlled sources has encouraged researchers and engineers to review the devices used for energy exchange and yield advanced techniques for active consumption of partial energy means. This energy requirement is fulfilled by the second analysis of thermodynamics. Entropy generation relates to the measure of eradication of productive energy. The heat transfer phenomenon initiated by its three main sources is the most feasible reason for such energy loss. Additionally, buoyancy and magnetic fields also contribute to entropy analysis. The minimization of entropy generation (EMG) in industrial procedures is among the novel concepts to augment the production of thermal systems. Several sources, such as heat transfer and viscous dissipation, are engaged for the formation of entropy. Additionally, entropy production establishes an augmentation in the power input in the power cycle for power consumption processes, whereas it contracts for power cycle outputs in a power-making system. Production in various systems can be upgraded with entropy generation and the Bejan number. Primarily, the first law of thermodynamics was employed for thermal efficiency in a system. Later, researchers testified that the second law investigation is as precise and significant compared to the first law.

Bejan [1] was the primary investigator who adapted the theory of entropy generation. He perceived that conductive and viscous irreversibilities are the two key sources of entropy generation. Analysis of heat and entropy for squeezing nonlinear fluid in the middle of the corresponding walls was discussed by Kaushik et al. [2]. Sheikholeslami et al. [3] offered an investigational study for entropy generation and exergy loss for nano-refrigerant compression progression. Afridi et al. [4] evaluated the combined impact of the Lorentz force and second law analysis on nanofluid dissipative flow about a curved medium. Nouri et al. [5] and Dormohammadi et al. [6] explored entropy analysis for nanofluid flows inside a channel. Convection and heat transfer of nanofluid with analysis of entropy inside a square cavity was inspected by Shermet et al. [7]. Entropy generation in unsteady magneto flow with a non-natural neural network and particle swarm optimization system was explored by Rashidi et al. [8]. Yongbo et al. [9] reported electro magnetohydro dynamics (EMHD) flow with entropy generation in a curved quadrilateral microchannel. Exploration of magneto slip flow under entropy generation on a revolving porous disk with adjustable properties was presented by Rashidi et al. [10]. Rehman et al. [11] proposed a study for the influence of radiation and thermal slip on rotating nanofluid with the entropy phenomenon. Researchers [12,13,14,15,16,17,18,19] have investigated the entropy generation on fluid flow with various physical effects.

Non-Newtonian fluids are generally involved in several organic states and have natural and manufacturing uses. Unlike Newtonian fluids, the non-Newtonian fluids own viscosity that is reliant on the shear rate or strain rate history. This compound feature makes non-Newtonian fluid valuable for various particular applications, e.g., coarse particles with transportation [20], while being relatively difficult to guess. In previous years, countless energy has been utilized to streamline the bond between the viscosity and the flow state of the non-Newtonian fluid, as it is crucial to many mathematical models to define this kind of fluid [21,22,23,24,25]. Several organic and natural fluids, such as body fluid and wet sand at the seaside, exhibit shear-thinning and shear-thickening rheological features. More recently, power-law fluid flow over a bluff frame has received considerable attention for some manufacturing products, such as paper-making, processing of food, and heat exchangers. Power-law fluid flow has a wide series of applications, including in food and chemical processing, pharmaceuticals, oil production, power generation devices, and heat exchangers. Power-law fluid [26,27,28,29], by amending its index, can be set to define the comprehensive activities of shear-dependent fluids. The power-law index indicates Newtonian fluid for *n* = 1, and otherwise characterizes shear-thinning (*n* < 1) or shear-thickening fluid (*n* > 1) correspondingly. Mohebbi et al. [30] deliberated on the power-law fluid flow inside two corresponding plates for various Reynolds numbers.

Flows of fluid over a permeable medium are of vital significance in power metallurgy, industrial filtration, petroleum technology, groundwater hydrology, ceramic engineering, etc. In the spirals of the geothermal region, water is an electrically conducting fluid due to peak temperature. The abundant power present in the soil’s layer in the geothermal regions has to be brought up to augment fuel output. This is an example to illustrate the flow past porous medium, taking the earth’s surface as a permeable bed. The temperature in the hot springs can be considered with the help of the energy equation. This temperature is useful to operate turbines through a magnetic field to produce electrical energy. In view of the aforementioned importance, investigators started working on porous layers with numerous geometries [31,32,33].

Several important and relevant kinds of literature on exponential stretching flows and different physical aspects, such as a non-uniform heat source and magenetohydro dyanmics (MHD), are listed in the references [34,35,36,37,38,39,40,41,42,43,44,45,46,47,48,49,50,51,52,53,54].

To the best of the authors’ knowledge, there seems to be no existing document on entropy analysis and a non-uniform heat source on power-law fluid with exponential stretching with heat flux conditions. Therefore, in this scientific report, our goal was to analyze the involvement of entropy generation with nonlinear power-law fluid above an exponential continuous moving surface. Both the shear-thinning and shear-thickening behaviors are discussed. The impact of a non-uniform heat source and suction/injection are also encouraged. The flow was controlled with the magnetohydrodynamics regime. The Runge–Kutta–Fehlberg method helps us to obtain the numerical explanation of the emerging equations. The model outcomes and comprehensive debates are provided in the form of results and discussions. In the conclusion, some key outcomes are also presented. The results obtained and presented in this article will portray that the optimal design and the efficient performance of a flow system or a thermally designed system can be enhanced by taking the suitable values of the physical parameters. This will allow us to reduce the effects of entropy generated within the system.

## 2. Definition of the Problem

**Figure 1:**
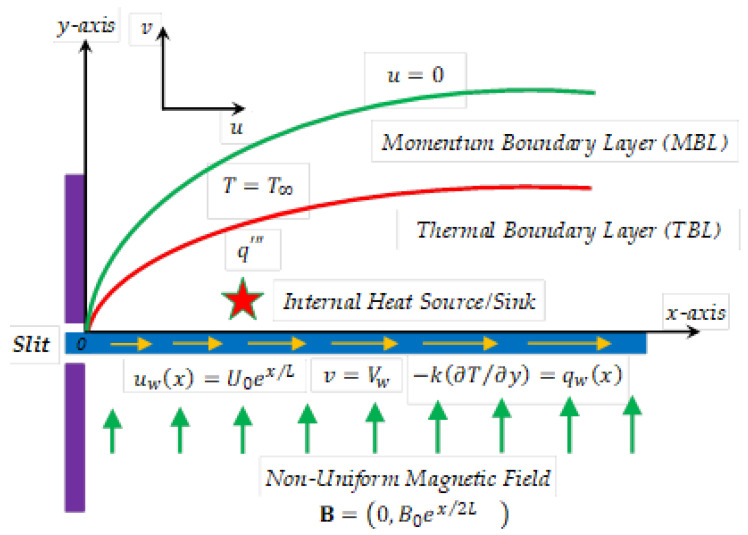
Geometry of the physical problem.

As described schematically in Figure 1, in this paper we propose to study the boundary layer flow and heat transfer characteristics of an incompressible electrically conducting power-law fluid passing over an exponential moving sheet with the presence of a variable heat source/sink $q^{\prime \prime \prime }$. With respect to assumptions, it is supposed that the stretching surface is non-uniformly heated via an imposed heat flux $q_{w}\left(x\right)=q_{0}\;e^{m\;x/L}$ and moved horizontally in the x-direction with a non-uniform velocity $u_{w}\left(x\right)=U_{0}\;e^{x/L}$, where x is the coordinate measured along the surface of the sheet. In addition, the occurring laminar flow of the non-Newtonian fluid is affected by the presence of horizontal stretching forces and the velocity $V_{w}$ of suction/injection at the permeable horizontal boundary y=0. An external applied magnetic field vector $B=\left(0,B_{0}\;e^{x/2L}\right)$ is functional perpendicular to the stretched surface. The total magnetic field exerted on the fluid flow can be regarded as the sum of the external magnetic field and the induced magnetic field. Physically, the magnetic Reynolds number is defined as the ratio of the advection term to the magnitude of the magnetic diffusion term. For similar flows, the magnetic Reynolds number is very small. Hence, the induced magnetic field can be neglected for the problem under consideration.

The energy equation is more prominent with the variable heat source/sink term. Moreover, an analysis for entropy generation and the Bejan number was also carried out to focus on the importance of the existing flow regime. Under these suppositions, the governing partial differential equations of the present boundary layer flow are stated as

(1) $$\frac{\partial u}{\partial x}+\frac{\partial v}{\partial y}=0,$$

(2) $$u\frac{\partial u}{\partial x}+v\frac{\partial u}{\partial y}=\frac{K}{\rho }\frac{\partial }{\partial y}\left({\left|\frac{\partial u}{\partial y}\right|}^{n-1}\frac{\partial u}{\partial y}\right)-\frac{\sigma B^{2}}{\rho }u,$$

(3) $$u\frac{\partial T}{\partial x}+v\frac{\partial T}{\partial y}=\alpha \frac{\partial^{2}T}{\partial y^{2}}+\frac{q^{\prime \prime \prime }}{\rho c_{p}\;},$$

with the following appropriate boundary conditions

(4) $$u=u_{w}\left(x\right),\;v=V_{w},\;-k\left(\frac{\partial T}{\partial y}\right)=q_{w}\left(x\right)=q_{0}\;e^{m\;x/L}\;\mathrm{At}\;y=0,\;u\to 0,\;T\to T_{\infty }\;\mathrm{as}\;y\to \infty$$

where K represents the consistency coefficient, L is a characteristic length, σ refers to the electrical conductivity, $U_{0}$, $B_{0}$, and $q_{0}$ are the reference velocity, magnetic field, and heat flux, respectively, and $V_{w}$ is the velocity of suction/injection, which is negative for suction $\left(i.e.,\;V_{w}<0\right)$ and positive for injection $\left(i.e.,\;V_{w}>0\right)$.

The particular problem can be specified in another straightforward form by using the following dimensionless variables

(5) η=y(ρ U0(2−n) 2KL)1n+1e(2−nn+1)(xL) , ψ=(2KLU02n−1ρ)1n+1e(2n−1n+1)(xL)f(η) , θ(η)=k(T−T∞ qwL)(Rex2 )1n+1.

Here, ${Re}_{x}$ is the generalized Reynolds number, where ${Re}_{x}=\rho \;u_{w}^{2-n}\;L^{n}/K$.

By virtue of the above considerations, the velocity components can be written in the form

(6) $$\left\{ \begin{array}{l} u=U_{0}\;e^{\frac{x}{L}}\;f^{\prime }\left(\eta \right),\\ v=-{\left(\frac{2KU_{0}^{2n-1}}{\rho L^{n}}\right)}^{\frac{1}{n+1}}\;e^{\left(\frac{2n-1}{n+1}\right)\frac{x}{L}}\left(\frac{2n-1}{n+1}f\left(\eta \right)+\frac{2-n}{n+1}\;\eta \;f^{\prime }\left(\eta \right)\right). \end{array}\right.$$

From Equation (6), it is obvious that the continuity equation (i.e., Equation (1)) is routinely satisfied.

The non-uniform heat source/sink $q^{\prime \prime \prime }$ term used in Equation (3) is defined as

(7) q‴=kL(ρ uw(2−n)2 K)2n+1[A*L qwk(Rex2)1n+1θ(0) f′+B*(T−T∞)],

where A^*^ and B^*^ are the coefficients of space and the temperature-dependent heat source/sink, respectively.

By making use of Equation (6), the boundary layer Equations (2) and (3) together with the boundary conditions in Equation (4) are reduced to

(8) $$n{\left|f^{\prime \prime }\right|}^{n-1}f^{\prime \prime \prime }+2\left(\frac{2n-1}{n+1}\right)ff^{\prime \prime }-2{f^{\prime }}^{2}-2M\;f^{\prime }=0,$$

(9) $$\theta^{\prime \prime }+\left(\frac{2n-1}{n+1}\right)\mathrm{Pr}f\theta^{\prime }+\left(\frac{3-(n+1)(m+1)}{n+1}\right)\mathrm{Pr}f^{\prime }\theta +A^{*}\theta \left(0\right){\;}_{}f^{\prime }+B^{*}\;\theta =0,$$

(10) $$f\left(0\right)=\left(\frac{n+1}{2n-1}\right){\;}_{}f_{w},\;f^{\prime }\left(0\right)=1,\;\theta^{\prime }\left(0\right)=-1,$$

(11) $$f^{\prime }\left(\infty \right)\to 0,\;\theta \left(\infty \right)\to 0.$$

Here, $M=\sigma B_{0}^{2}L/\left(\rho \;U_{0}\right)$ is the magnetic parameter, Pr=(uwL/α)(Rex/2)−2n+1 is the generalized Prandtl number, $\alpha =k/\left(\rho c_{p}\right)$ is the thermal diffusivity, and $f_{w}=-\left(V_{w}/u_{w}\right){\left({Re}_{x}/2\right)}^{-\frac{1}{n+1}}$ is the suction/injection parameter.

## 3. Relevant Physical Measures

Keeping in mind the above considerations, the local skin friction $C_{fx}$ coefficient and the heat transfer coefficient $Nu_{x}$ for the concerned problem are given by

(12) {(Rex2)1n+1Cfx =|f″(0)|n−1f″(0),(Rex2)−1n+1 Nux =1θ(0).

## 4. Entropy Generation and Bejan Number

By employing the second law of thermodynamics in the present MHD boundary layer flow problem, the volumetric entropy generation is defined locally by [16,17,18,19]

(13) $${{\dot{S}}^{\prime \prime \prime }}_{gen}=k{\left(\frac{\nabla T}{T}\right)}^{2}+\frac{\;\mu_{}\Psi \;}{T}+\frac{J^{2}}{\sigma_{}T}.$$

Where ∇T refers to the temperature gradient vector, Ψ denotes the viscous dissipation function, and J represents the current density vector, such that

(14) J=σ(V×B).

Consequently, the volumetric entropy generation ${{\dot{S}}^{\prime \prime \prime }}_{gen}$ can take the following form

(15) $${{\dot{S}}^{\prime \prime \prime }}_{gen}={{\dot{S}}^{\prime \prime \prime }}_{h}+{{\dot{S}}^{\prime \prime \prime }}_{f}+{{\dot{S}}^{\prime \prime \prime }}_{m},$$

in which

(16) $${{\dot{S}}^{\prime \prime \prime }}_{h}=\frac{k}{T_{\infty }^{2}}{\left(\frac{\partial T}{\partial y}\right)}^{2},$$

(17) $${{\dot{S}}^{\prime \prime \prime }}_{f}=\frac{K}{T_{\infty }}{\left|\frac{\partial u}{\partial y}\right|}^{n-1}{\left(\frac{\partial u}{\partial y}\right)}^{2},$$

(18) $${{\dot{S}}^{\prime \prime \prime }}_{m}=\frac{\sigma B^{2}}{T_{\infty }}\;u^{2}.$$

Therefore, Equation (15) becomes

(19) $${{\dot{S}}^{\prime \prime \prime }}_{gen}=\frac{k}{T_{\infty }^{2}}{\left(\frac{\partial T}{\partial y}\right)}^{2}+\frac{K}{T_{\infty }}{\left|\frac{\partial u}{\partial y}\right|}^{n-1}{\left(\frac{\partial u}{\partial y}\right)}^{2}+\frac{\sigma B^{2}}{T_{\infty }}\;u^{2}.$$

The dimensionless form of Equation (19) is called the entropy generation and symbolized by $N_{s}$. This thermodynamic quantity is given by

(20) $$N_{s}=\frac{{{\dot{S}}^{\prime \prime \prime }}_{gen}}{{{\dot{S}}^{\prime \prime \prime }}_{0}}={\left(\frac{{Re}_{x}}{2}\right)}^{\frac{2}{n+1}}\;{\theta^{\prime }}^{2}+\frac{Re\;Br}{2\;\Omega }\left({\left|f^{\prime \prime }\right|}^{n-1}{\left(f^{\prime \prime }\right)}^{2}+2M\;{f^{\prime }}^{2}\right),$$

where ${{\dot{S}}^{\prime \prime \prime }}_{0}=k\;\Delta T/\left(L^{2}\;T_{\infty }^{2}\right)$ is a characteristic entropy generation, $Re=\rho \;u_{w}L/K$ is the Reynolds number, $Br=U_{0}^{2}K/\left(k\;T_{\infty }\right)$ is the Brikman number, and $\Omega =\Delta T/T_{\infty }$ is the temperature difference parameter.

Also, the Bejan number Be is introduced to check the domination of the heat transfer part over the fluid friction part along with MHD. This dimensionless quantity is defined as

(21) $$B_{e}=\frac{{{\dot{S}}^{\prime \prime \prime }}_{h}}{{{\dot{S}}^{\prime \prime \prime }}_{gen}}=\frac{1}{1+\Phi },$$

where $\Phi =\left({{\dot{S}}^{\prime \prime \prime }}_{f}+{{\dot{S}}^{\prime \prime \prime }}_{m}\right)/{{\dot{S}}^{\prime \prime \prime }}_{h}$ is the irreversibility quotient.

From Equation (21), it is observed that the heat transfer is higher when 0≤Φ<1, whereas the other parts can dominate when Φ>1. Also, if Φ=1, the influence of both parts is of the same magnitude. As proven above, the Bejan number Be ranges from 0 to 1 (see reference [55] for more details). In addition, the heat transfer irreversibility can dominate when Be=1. Moreover, the frictional and magnetic irreversibilities became more prominent when Be = 0. Furthermore, the heat transfer and the other parts have an equal contribution when Be=0.5. Moreover, the manner of the Bejan number Be is premeditated for the optimal values of the constraints for which the entropy generation takes its lowest value.

## 5. Solution Methodology and Validation of Results

The nonlinear differential Equations (8) and (9) along with their corresponding boundary conditions (10) and (11) constitute a two-point boundary value problem. These differential equations are solved numerically by utilizing the Shooting Method (SM). For this purpose, the resulting differential system arising from Equations (8)–(11) is converted into an Initial Value Problem. By applying this method, Equations (8) and (9) can be reduced to a system of first-order ordinary differential equations by setting

(22) $$\left(h_{1},h_{2},h_{3},h_{4},h_{5}\right)=\left(f{,}_{}f^{\prime }{,}_{}f^{\prime \prime },\;\theta_{},{\;}_{}\theta^{\prime }\right).$$

After introducing Equation (22) into Equations (8) and (9), we obtain the following reduced differential equations

(23) $$n{\left|h_{3}\right|}^{n-1}h_{3}{}^{\prime }+2\left(\frac{2n-1}{n+1}\right)h_{1}\;h_{3}-2\;h_{1}^{2}-2M\;h_{2}=0,$$

(24) $$h_{5}{}^{\prime }+\left(\frac{2n-1}{n+1}\right)\mathrm{Pr}h_{1}\;h_{5}+\left(\frac{3-(n+1)(m+1)}{n+1}\right)\mathrm{Pr}h_{2}\;h_{4}+A^{*}\;h_{4}\left(0\right)\;h_{2}+B^{*}\;h_{4}=0,$$

(25) $$h_{1}(0)={\left(\frac{n+1}{2n-1}\right)}_{}f_{w},\;h_{2}(0)=1,\;h_{3}(0)=s_{1},\;h_{4}(0)=s_{2},\;h_{5}\left(0\right)=-1.$$

In this problem, the SM is used as a powerful technique to guess the missing initial conditions $s_{1}$ and $s_{2}$ by means of an iterative process until the boundary conditions are satisfied, in such a way that the reduced Equations (23)–(25) were integrated numerically by utilizing the Runge–Kutta–Fehlberg method and taking Δη=0.001 as the best step size. Then, the computed values of $f^{\prime }\left(\eta \right)$ and θ(η) at $\eta_{\infty }$ were compared with the given boundary conditions $f^{\prime }\left(\eta_{\infty }\right)=0$ and $\theta \left(\eta_{\infty }\right)=0$. The guessed values of $f^{\prime \prime }(0)$ and θ(0) were refined with the help of the Newton–Raphson Method to give a better approximation for the desired solutions. The iterative process was repeated until we acquired results with an accuracy level of about of $10^{-6}$.

In order to verify the precise running of the program, the numeric values of the wall shear stress $f^{\prime \prime }\left(0\right)$ for the viscous fluid case were related to the findings of Magyari and Keller [56], Elbashbeshy [57], Sahoo and Poncet [58], Mukhopadhyay et al. [59], and Sajid and Hayat [60] in their pioneering studies. As expected, it was found that our results are in good agreement with those in the existing literature [56,57,58,59,60] (see Table 1).

## 6. Results and Discussion

**Figure 2:**
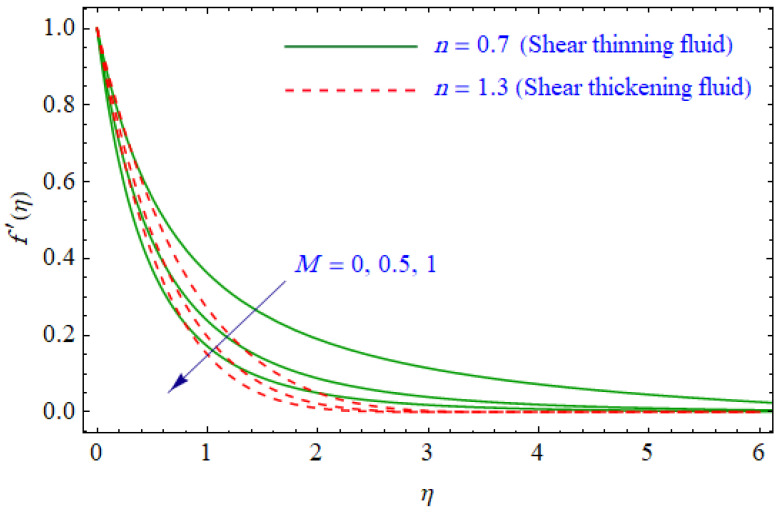
Profiles of $f^{\prime }(\eta )$ for various values of M, when n=0.7 and n=1.3.

**Figure 3:**
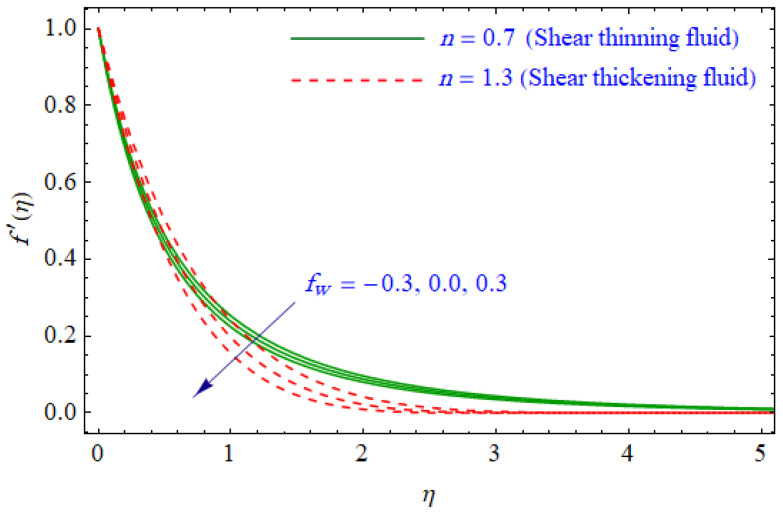
Profiles of $f^{\prime }(\eta )$ for various values of $f_{w}$, when n=0.7 and n=1.3.

**Figure 4:**
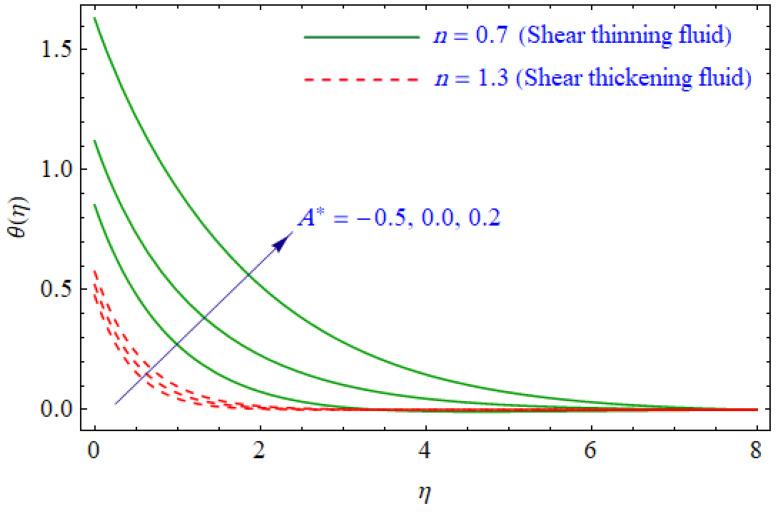
Profiles of θ(η) for various values of $A^{*}$, when n=0.7 and n=1.3.

**Figure 5:**
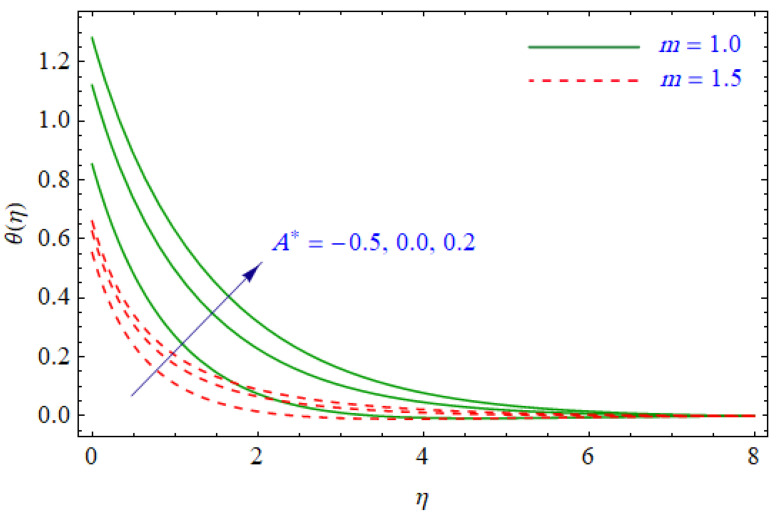
Profiles of θ(η) for various values of $A^{*}$, when m=1.0 and m=1.5.

**Figure 6:**
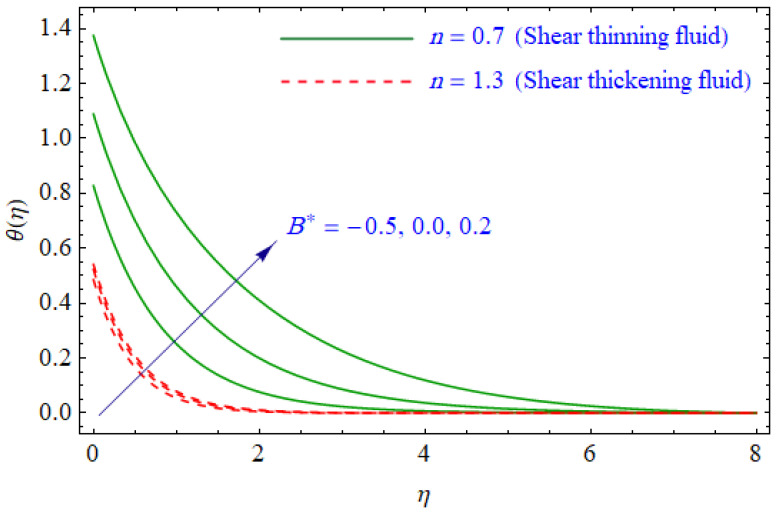
Profiles of θ(η) for various values of $B^{*}$, when n=0.7 and n=1.3.

**Figure 7:**
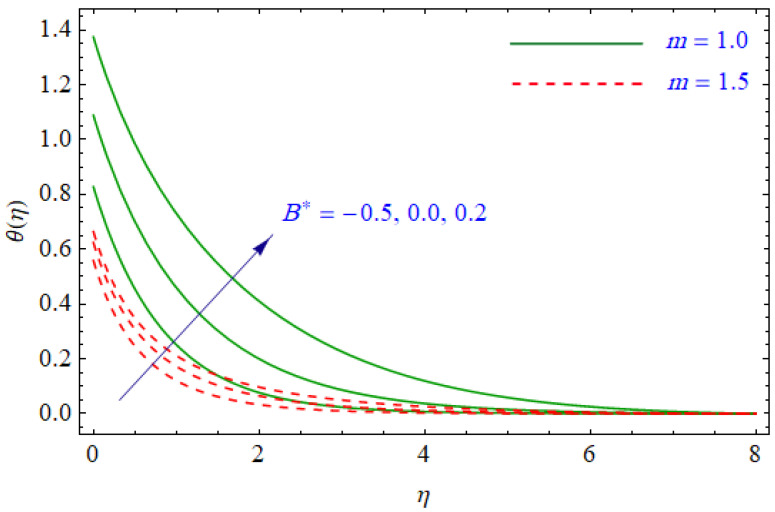
Profiles of θ(η) for various values of $B^{*}$, when m=1.0 and m=1.5.

**Figure 8:**
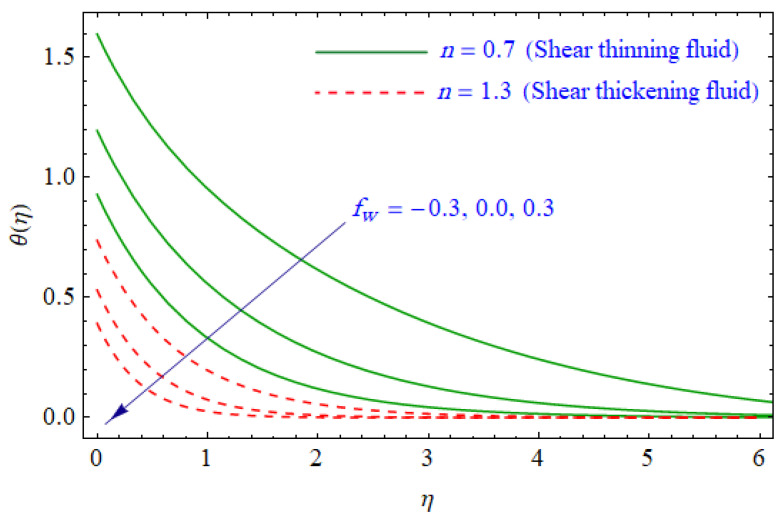
Profiles of θ(η) for various values of $f_{w}$, when n=0.7 and n=1.3.

**Figure 9:**
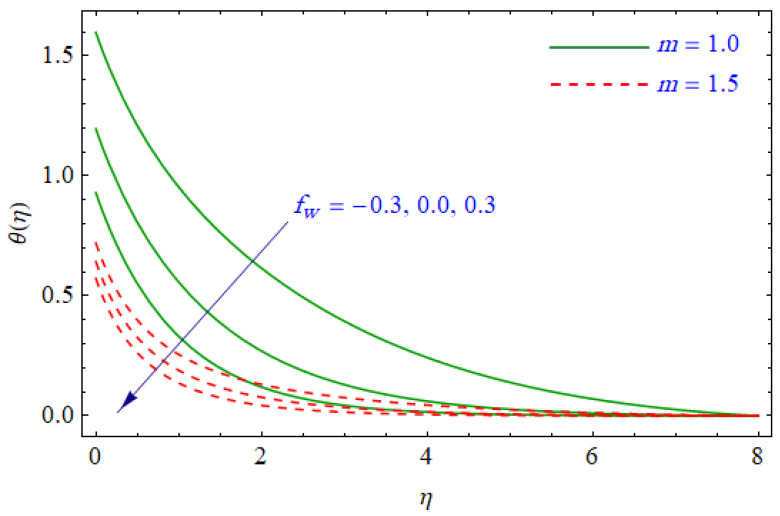
Profiles of θ(η) for various values of $f_{w}$, when m=1.0 and m=1.5.

**Figure 10:**
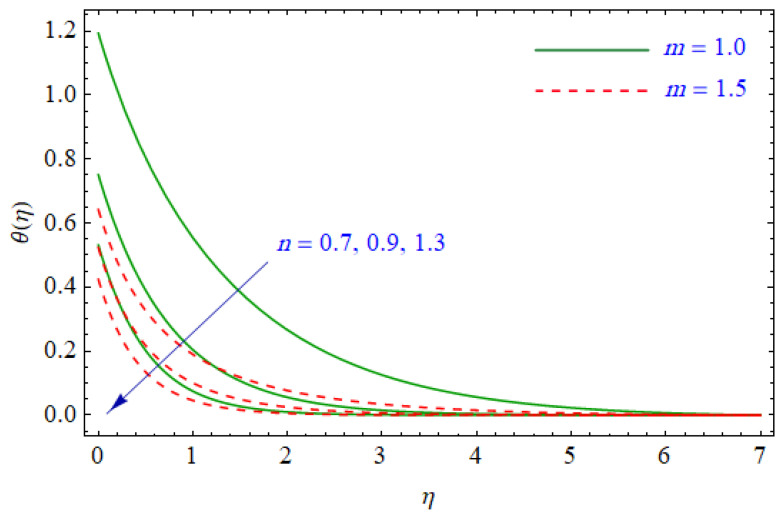
Profiles of θ(η) for various values of n, when m=1.0 and m=1.5.

**Figure 11:**
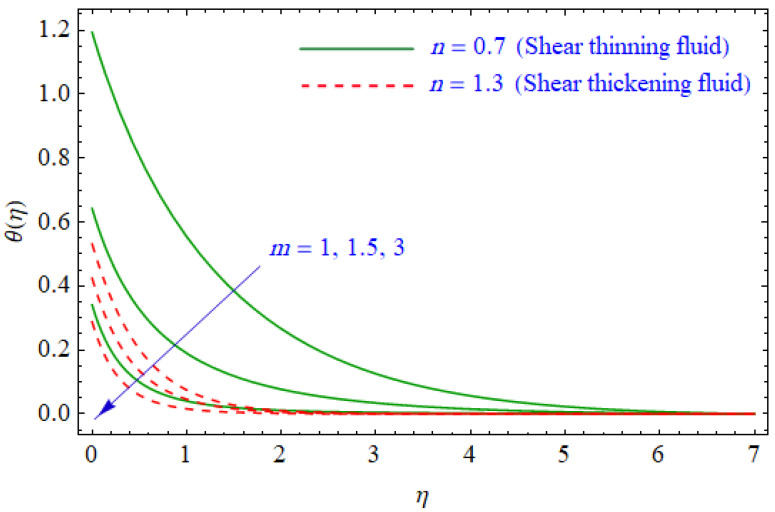
Profiles of θ(η) for various values of m, when n=0.7 and n=1.3.

**Figure 12:**
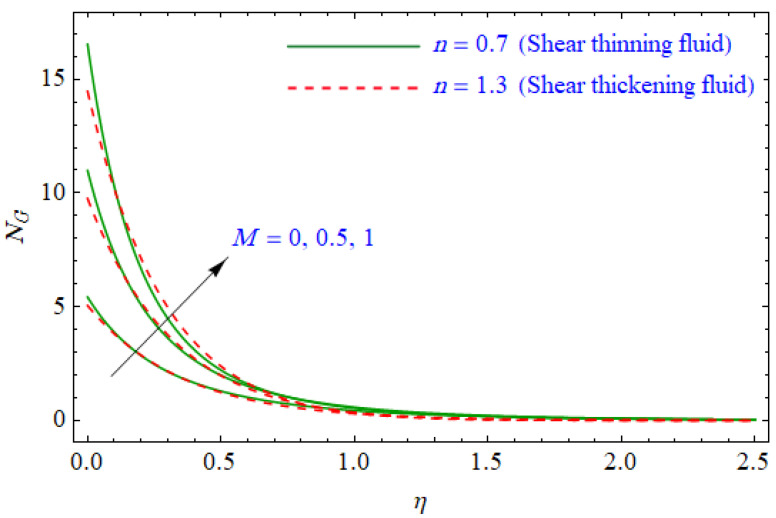
Profiles of $N_{G}(\eta )$ for various values of M, when n=0.7 and n=1.3.

**Figure 13:**
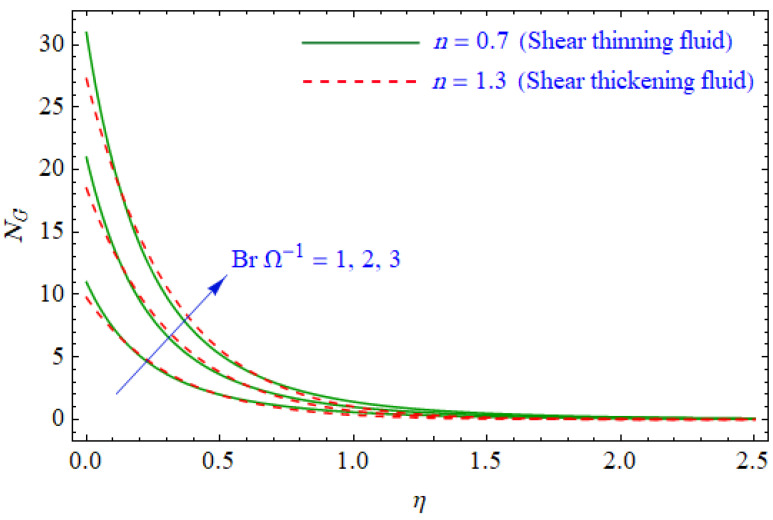
Profiles of $N_{G}(\eta )$ for various values of $Br\Omega^{-1}$, when n=0.7 and n=1.3.

**Figure 14:**
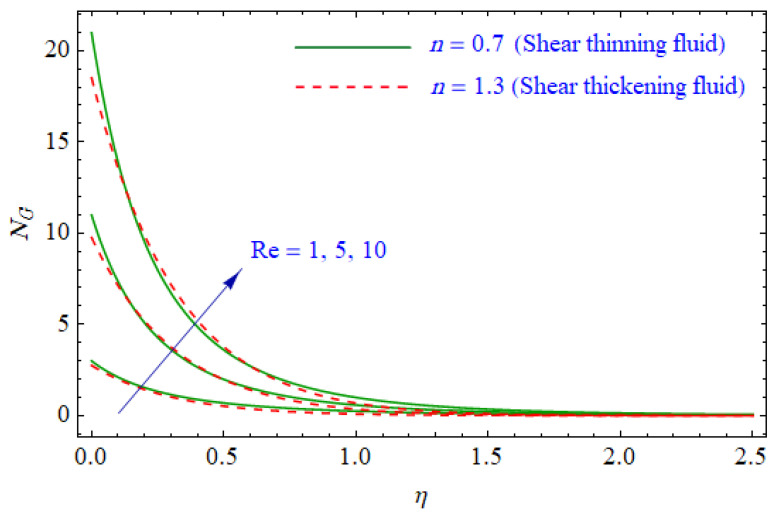
Profiles of $N_{G}(\eta )$ for various values of Re, when n=0.7 and n=1.3.

**Figure 15:**
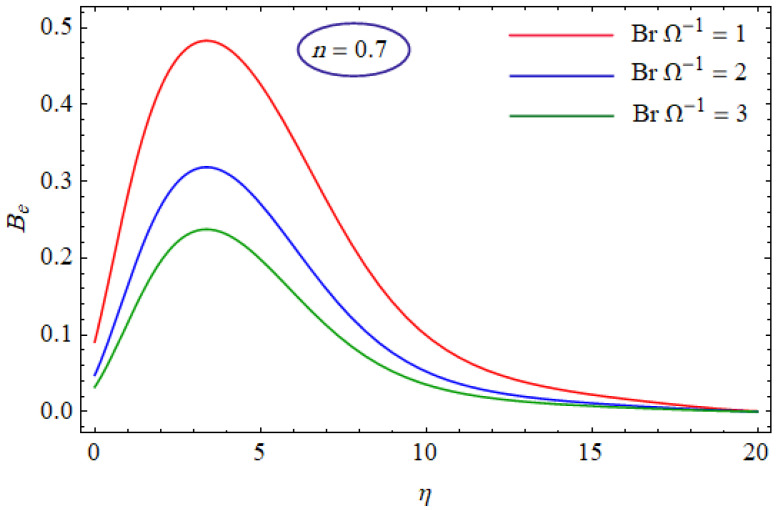
Profiles of Be(η) for various values of $Br\Omega^{-1}$, when n=0.7.

**Figure 16:**
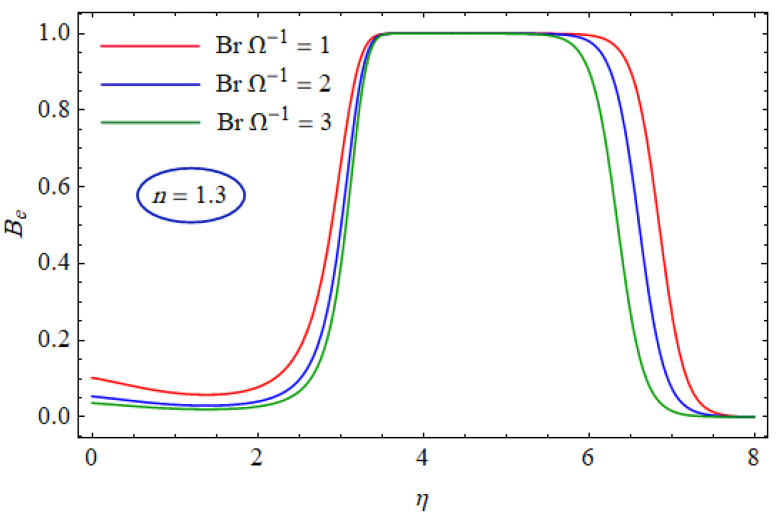
Profiles of Be(η) for various values of $Br\Omega^{-1}$, when n=1.3.

**Figure 17:**
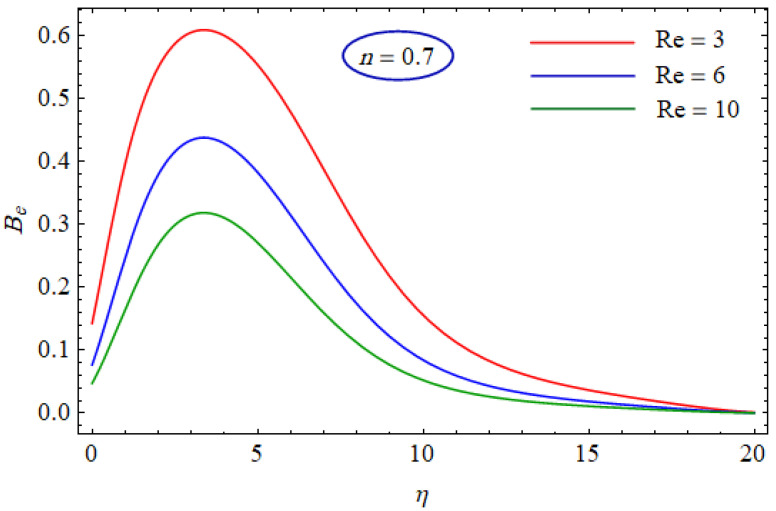
Profiles of Be(η) for various values of Re, when n=0.7.

**Figure 18:**
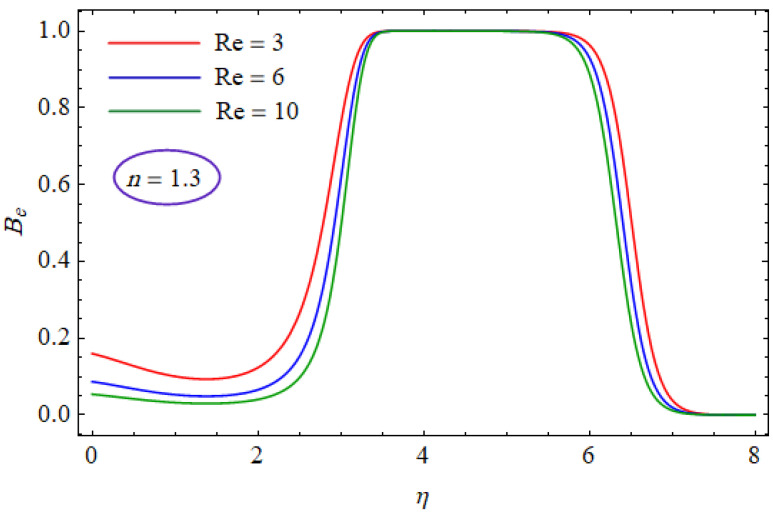
Profiles of Be(η) for various values of Re, when n=1.3.

**Figure 19:**
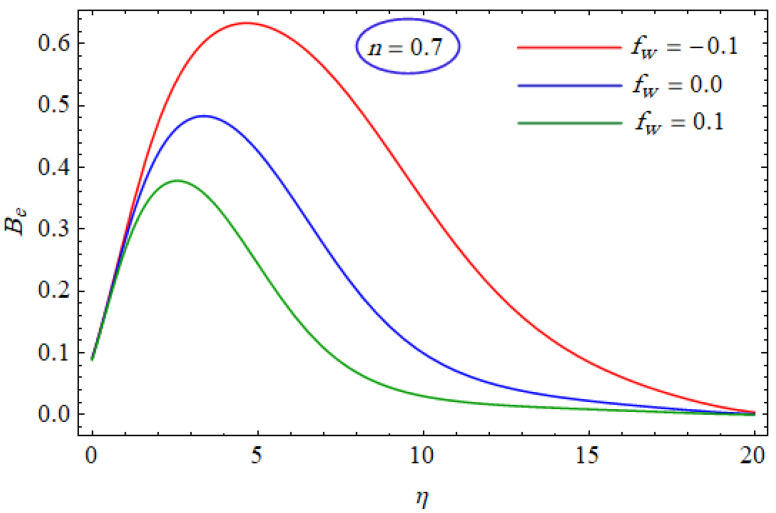
Profiles of Be(η) for various values of $f_{w}$, when n=0.7.

**Figure 20:**
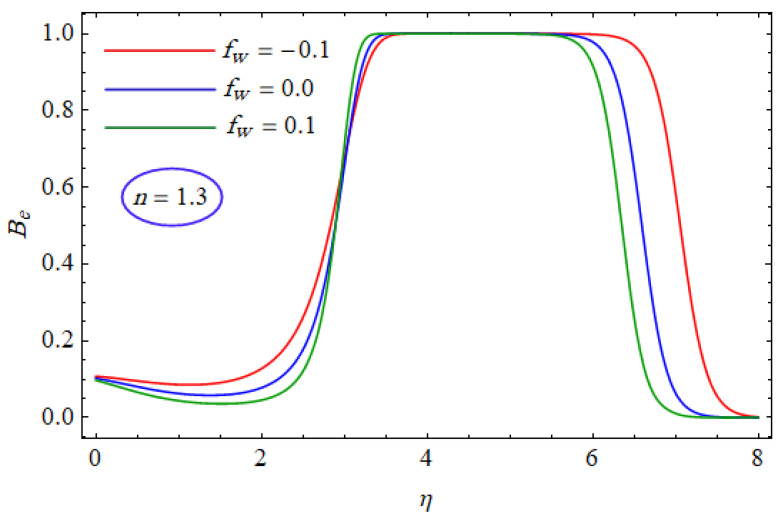
Profiles of Be(η) for various values of $f_{w}$, when n=1.3.

**Figure 21:**
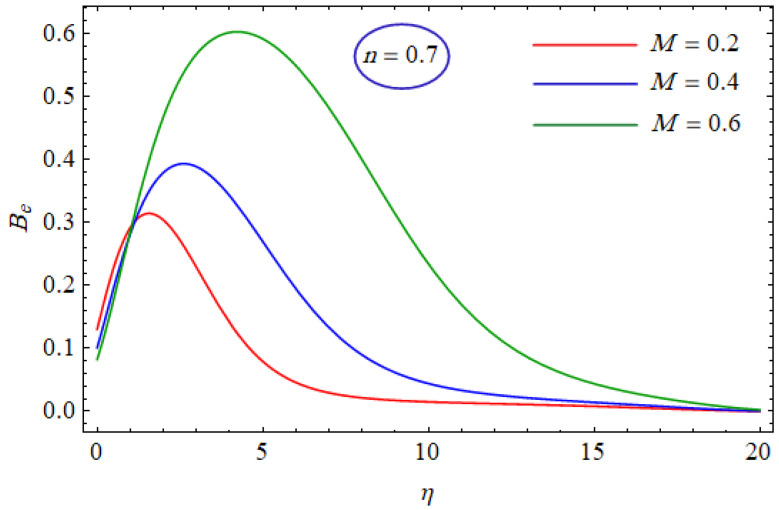
Profiles of Be(η) for various values of M, when n=0.7.

**Figure 22:**
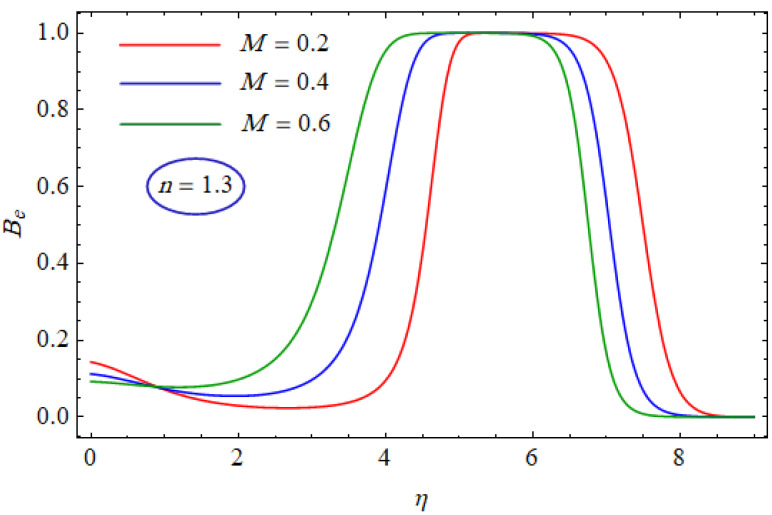
Profiles of Be(η) for various values of M, when n=1.3.

This segment of the present work highlights the impression of related variables on exponential flow of non-Newtonian power-law fluid. Figure 2, Figure 3, Figure 4, Figure 5, Figure 6, Figure 7, Figure 8, Figure 9, Figure 10, Figure 11, Figure 12, Figure 13, Figure 14, Figure 15, Figure 16, Figure 17, Figure 18, Figure 19, Figure 20, Figure 21 and Figure 22 are plotted to explain the variations of velocity, temperature, entropy analysis, the Bejan number, and flow and heat fluxes in both cases of shear thinning and shear thickening. Figure 2 is dedicated to observing the effect of the magnetic parameter (M= 0, 0.5, 1)  on the velocity of fluid. It was found that the resistive Lorentz force due to a magnetic field reduces the motion of the fluid. The magnitude of momentum boundary layer was higher for the shear thickening case, i.e., n=1.3. Figure 3 discloses the effects of suction/injection $\left(f_{w}=\;-0.3,\;0.0,\;0.3\right)\;$ for velocity with n=0.7 and n=1.3. It was observed that the thickness of the boundary layer shrinks extra-rapidly for the n=1.3  case. The impact of internal heat source parameters $\left(A^{*}=-0.5,\;0,\;0.5\right)$ and $\;\left(B^{*}=-0.5,\;0,\;0.2\right)$ with (n=0.7, 1.3) and (m=1, 1.5) is sketched in Figure 4, Figure 5, Figure 6 and Figure 7, respectively. The temperature of the considered fluid increased in all cases and the enhancement of the thermal boundary layer was maximum for n=0.7 and m=1, respectively. Figure 8 and Figure 9 are presented to display the nature of the temperature for suction/injection $\left(f_{w}=\;-0.3,\;0.0,\;0.3\right)\;$ with (n=0.7, 1.3) and (m=1, 1.5), respectively. It is clear that this parameter is an inverse function of temperature and there is less variation in magnitude for the n=1.3 and m=1.5 case. The power-law index  (n=0.7, 0.9, 1.3) causes a decrement in temperature (see Figure 10). Figure 11 elucidates the effect of the heat flux index  (m=1, 1.5, 3) on the temperature profile. Obviously, the thermal boundary layer thickness decreases with a higher m. The impact of a magnetic constraint (M= 0, 0.5, 1)  on entropy is shown in Figure 12. It is depicted that the Lorentz force created by the magnetic field augmented the entropy of the system. Evidently, M is very sensitive to entropy generation augmentation. This is due to the fact that the magnetized fluid forced the dissipative heat energy to thermal diffusion. Figure 13 and Figure 14 were developed to study the variation of entropy generation for the group parameter $\left(Br\Omega^{-1}=1,\;2,\;3\right)$ and the Reynolds number (Re=3,5, 10)  with  n=0.7, 1.3, respectively. Both parameters are directly proportional to the entropy analysis and the magnitude is almost similar for both n=0.7, 1.3 cases.

Essentially, the Brinkman number estimates the heat that is discharged by viscous heating in connection with heat exchange because of conduction of particles. Close to the sheet, the viscous impacts delivered a lower measure of heat when contrasted with exchange of heat strength by particle conduction. The large measure of heat that developed between liquid particles is a reason for entropy improvement.

In Figure 15 and Figure 16, the Bejan number for several values of the group parameter $\left(Br\Omega^{-1}=1,\;2,\;3\right)$ with n=0.7 and n= 1.3 is explored, respectively. It was perceived that irreversibility of fluid friction attains control near the cold permeable surface (Be shrinkages) as the group parameter rises. On the heated permeable surface, fluid friction irreversibility showed entire control over the irreversibility of heat transfer irrespective of the range of the group parameter involved. Figure 17 and Figure 18 delineate the variation of the Bejan number for the Reynolds number (Re=1, 5, 10) for both cases, i.e., shear thickening and shear thinning. The Bejan number decreased when the Re increased. The variation is very interesting from a physical point of view for n= 1.3, as reducing the Bejan number indicated the increasing dominance of fluid friction over the heat transfer irreversibility. The effect of suction/injection $\left(f_{w}=\;-0.3,\;0.0,\;0.3\right)$ with n=0.7 and 1.3 is portrayed in Figure 19 and Figure 20, respectively. It was shown that, as suction escalates on the cold absorbent surface, there was improved control of heat transfer irreversibility and when injection rose on the cold permeable plate, the domination of heat transfer irreversibility on fluid friction irreversibility declined. It was found that, for the shear thickening case, near the centerline of the surface, there was complete supremacy of heat transfer irreversibility (Be = 1) for fluctuating values of $f_{w}$ while there was entire authority of fluid friction irreversibility (Be = 0) near the isothermally heated permeable surface. The impact of the magnetic parameter (M= 0, 0.5, 1) with n=0.7 and 1.3 is plotted in Figure 21 and Figure 22, respectively. For n=0.7, initially the Bejan number decreased and after η=1 the variation was the opposite, i.e., increasing. The variation of the Bejan number was observed to reach the maximum from η=4.5 to 6.5 and then decline again for n=1.3. It was also observed that, near the surface of the plate, the influences of magnetic and viscous irreversibilities were dominant compared with the heat transfer irreversibility (i.e., Be<0.5).

Table 2 and Table 3 were constructed to highlight the impression of some proper parameters on the skin friction coefficient and the Nusselt number. The two cases, i.e., shear thinning and shear thickening, are reflected correspondingly. It was noticed that the magnetic parameter (M=0, 0.5, 1.0) produced the minimum flow flux and heat transfer rate at the wall for both considered cases. The skin friction coefficient showed shrinking behavior for suction/injection $\left(f_{w}=\;-0.3,\;0.0,\;0.3\right)$, while increasing the heat transfer rate. The heat flux index and internal heat source parameters had negligible influence on the skin friction coefficient as deliberated by both the tables. Moreover, the Nusselt number reduced with increasing heat flux index but the variation was opposite for the internal heat source.

## 7. Conclusions

Heat transfer and second law analysis for non-Newtonian power-law fluid over an exponential continuous moving surface were studied using the Runge–Kutta–Fehlberg method. This analysis was conducted with the appropriate parameters for the following choices: magnetic parameter, Reynolds number, group parameter, suction/injection, internal heat source, power-law index, and heat flux index.

The significant conclusions are as follows:

- The Bejan number expressively decreased and the total entropy generation was augmented with the growing Reynolds number.
- The quality of energy reduces, i.e., entropy generation is enhanced, with large magnetic and group parameters.
- The velocity of the power-law fluid varies indirectly with the suction/injection parameter.
- The heat transfer rate decay under the presence of an internal heat source and magnetic field was established.
- Thermal boundary layer decreases with an increasing heat flux index during the phenomenon.

It is expected that the current outcomes will shed light on several physical features of this problem and will assist as a motivation for more experimental works in the area of entropy generation and irreversibility analysis.

## Figures and Tables

**Table 1:** Comparison between our findings and those of the existing literature results [56,57,58,59,60], in the case where n=1 and M=0.

Existing Results for $f^{\prime \prime }\left(0\right)$					Present Result for $f^{\prime \prime }\left(0\right)$
[56]	[57]	[58]	[59]	[60]	SRKFM
−1.28180	−1.28181	−1.281811	−1.281812	−1.28181	−1.2818098

**Table 2:** Flow and heat transfer characteristics of a shear thinning fluid, for various values of $M,\;f_{w},\;m\;,\;A^{*}$, and $B^{*}$, when Pr=5 and n=0.7. Bold number shows the respective involved parameter while keeping remaining constant.

*M*	$f_{w}$	m	$A^{*}$	$B^{*}$	(Rex2)1n+1Cfx	(Rex2)−1n+1 Nux
**0.0**					−1.264728	0.957247
**0.5**	0.0	1.0	0.1	0.1	−1.570997	0.836066
**1.0**					−1.808090	0.731285
	**−0.3**				−1.513345	0.625733
0.5	**0.1**	1.0	0.1	0.1	−1.590886	0.913095
	**0.3**				−1.631743	1.075468
		**0.8**			−1.570988	0.441335
0.5	0.0	**1.5**	0.1	0.1	−1.571019	1.556417
		**3.0**			−1.571019	2.930572
			**−0.5**		−1.570997	1.171080
0.5	0.0	0.1	**0.0**	0.1	−1.570997	0.891902
			**0.5**		−1.570997	0.441335
				**−0.5**	−1.570997	1.208040
0.5	0.0	0.1	0.1	**0.0**	−1.570997	0.918485
				**0.2**	−1.570997	0.727479

**Table 3:** Flow and heat transfer characteristics of a shear thickening fluid, for various values of $M,\;f_{w},\;m\;,\;A^{*}$, and $B^{*}$, when Pr=5 and n=1.3. Bold number shows the respective involved parameter while keeping remaining constant.

*M*	$f_{w}$	m	$A^{*}$	$B^{*}$	(Rex2)1n+1Cfx	(Rex2)−1n+1 Nux
**0.0**					−1.313498	1.956119
**0.5**	0.0	1.0	0.1	0.1	−1.679224	1.883365
**1.0**					−1.992612	1.822293
	**−0.3**				−1.470333	1.354459
0.5	**0.1**	1.0	0.1	0.1	−1.754932	2.089481
	**0.3**				−1.915439	2.546558
		**0.8**			−1.679185	1.668265
0.5	0.0	**1.5**	0.1	0.1	−1.679185	2.355932
		**3.0**			−1.679185	3.474166
			**−0.5**		−1.679185	2.099785
0.5	0.0	0.1	**0.0**	0.1	−1.679185	1.917798
			**0.5**		−1.679185	1.735811
				**−0.5**	−1.679185	2.060122
0.5	0.0	0.1	0.1	**0.0**	−1.679185	1.913069
				**0.2**	−1.679185	1.848783
